# Linguistic methods in healthcare application and COVID-19 variants classification

**DOI:** 10.1007/s00521-021-06286-y

**Published:** 2021-07-06

**Authors:** Marek R. Ogiela, Urszula Ogiela

**Affiliations:** 1 Cryptography and Cognitive Informatics Laboratory, AGH University of Science and Technology, 30 Mickiewicza Ave, 30-059 Kraków, Poland; 2 Pedagogical University of Krakow, Podchorążych 2 St, 30-084 Kraków, Poland

**Keywords:** Mathematical linguistics, Healthcare application, Formal grammars, Pandemic prevention

## Abstract

One of the most important goals of modern medicine is prevention against pandemic and civilization diseases. For such tasks, advanced IT infrastructures and intelligent AI systems are used, which allow supporting patients’ diagnosis and treatment. In our research, we also try to define efficient tools for coronavirus classification, especially using mathematical linguistic methods. This paper presents the ways of application of linguistics techniques in supporting effective management of medical data obtained during coronavirus treatments, and possibilities of application of such methods in classification of different variants of the coronaviruses detected for particular patients. Currently, several types of coronavirus are distinguished, which are characterized by differences in their RNA structure, which in turn causes an increase in the rate of mutation and infection with these viruses.

## Introduction

One of the main challenges of modern medicine is the fight against pandemic and civilization diseases. In such activities, computer methods and systems play a huge role in supporting all diagnostic processes, and even managing hospital infrastructure or staff [1]. Many artificial intelligence algorithms operating in IoT environments capture critical data and support decision optimization through fast and intelligent data analysis [2, 3]. Modern medicine is patient-oriented thanks to artificial intelligence algorithms, which also enable extensive monitoring of his vital functions, as well as treatment, rehabilitation and remote medical consultations. Now, despite the significant development of the level of medical services and the use of systems supporting processes in healthcare, there are new significant challenges related to the coronavirus pandemic (COVID SARS-CoV-2) [4]. In this context, diagnostic support systems and patient treatment must also be oriented towards new application areas related to the detection, treatment and rehabilitation of patients affected by the COVID virus [5].

This paper will present new possibilities of using mathematical linguistics techniques in supporting such medical processes and will indicate new areas of healthcare, in which such techniques will play an important role [6]. In particular, the systems that allow for effective management of medical data obtained in the fight against coronavirus will be described, and the idea of using such methods in the classification of types (mutations) of the virus that has been detected in a specific patient will be presented. Currently, several types of coronavirus are distinguished, which are characterized by differences in their structural structure, which in turn causes an increase in the rate of mutation and infection with these viruses.

## Linguistic methods in medical application

Linguistic methods focus on analyzing various types of formal grammars and refer directly to cognitive informatics. Mathematical linguistic formalisms were first developed by N. Chomsky for modeling natural languages [6]. He proposed the four classes of formal grammars, i.e., phrase structure grammars, context grammars, context-free grammars, and regular grammars.

Now we can observe the constantly growing opportunities for application of formal grammars especially in creation of cognitive information systems, image understanding methods and linguistic cryptographic procedures. Such formalisms are also applicable in creations of modern algorithms for information sharing [7, 8].

Below is presented basic definition of formal grammars, which can be used in the creation of linguistic threshold schemes [8].

### Definition

A formal grammar (a grammar) is a quadruple: $G = \left( {N,T,PS,STS} \right)$, where *N*—set of non-terminal symbols; *T*—set of terminal symbols; *PS*—set of rewriting rules (productions) in the form: $X \to a,$ where $X \in N,$ $a \to N \cup T$; *STS*—a start symbol of the grammar, and $STS \in N$

This definition is a general definition of all sequential grammars, which after introducing additional constraints for rewriting rules can define particular grammar classes introduced by N. Chomsky.

## Linguistic threshold schemes in healthcare security and data management

The purposes of this section are to define a new extension of secret sharing techniques, called linguistic threshold schemes, where security will be based on the application of mathematical linguistic formalisms.

**Fig. 1:**
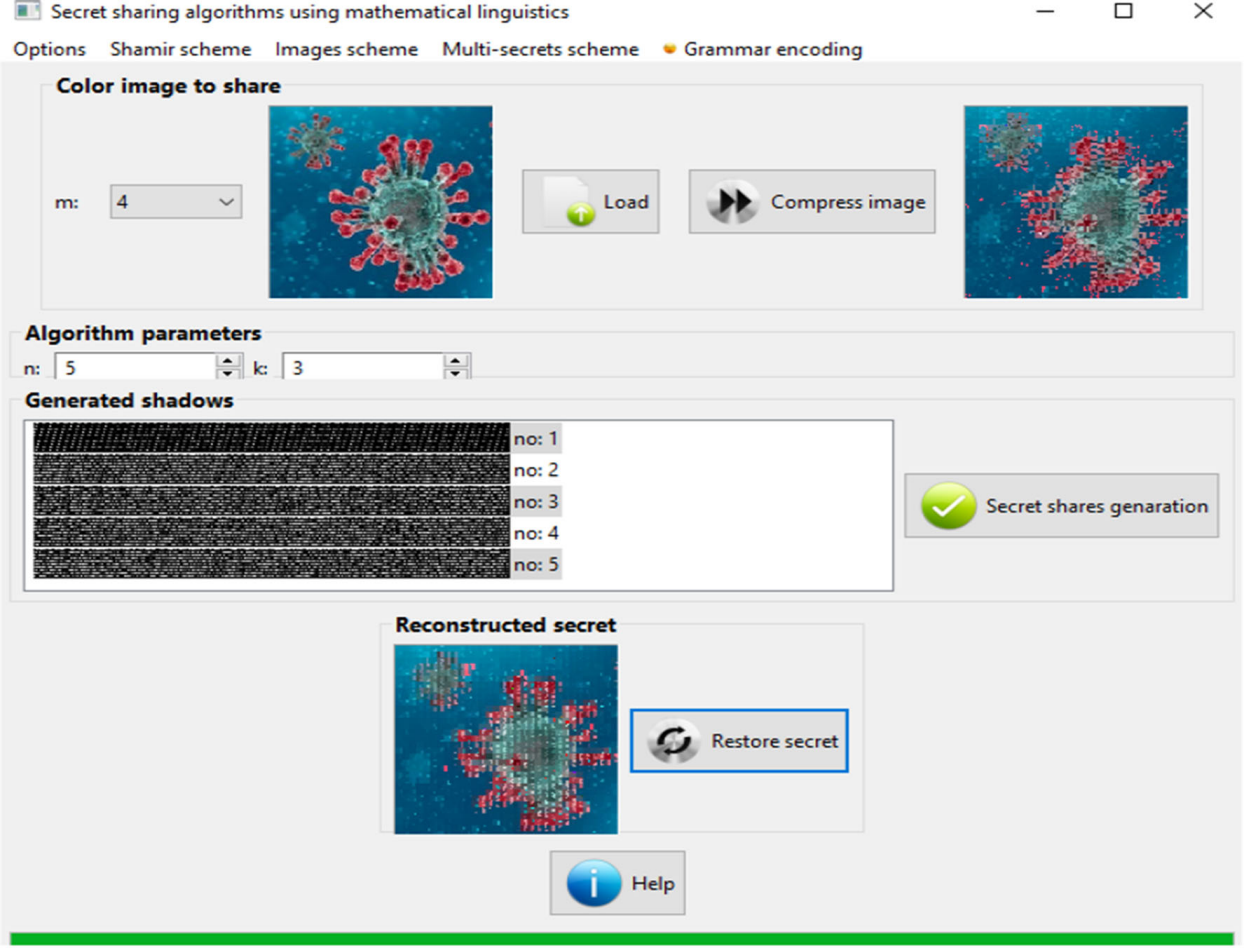
An example of image threshold sharing using (3,5)—threshold schemes

Computer techniques used for secret information division are a new field of IT application. They allow to split strategic or medical data in such manner that it can be distributed among certain group of authorized persons or users, who after collecting them can reveal the original message (Fig. 1).

In Fig. 1 is presented an example of image division using threshold procedure. An original image and its compressed version are presented in the boxes located in the top area of this screenshot. Below images are visible threshold parameters (values 5 and 3), which determine the quantity of generated visual parts. Obtained visual shares are visible in the form of gray-shadow bars located in the “generated shadows” window. The last image from the bottom presents the reconstructed version of original visual pattern.

There are two main groups of secret division algorithms, i.e., secret splitting and secret sharing. Both types allow to generate any number of secret parts of the information, but for its reconstruction secret splitting procedures require the whole number of parts, but in secret sharing it is necessary to collect the smaller number of parts for secret reconstruction [9].

More universal are secret sharing methods, which allow to divide data for any number of parts depending only on threshold values.

**Fig. 2:**
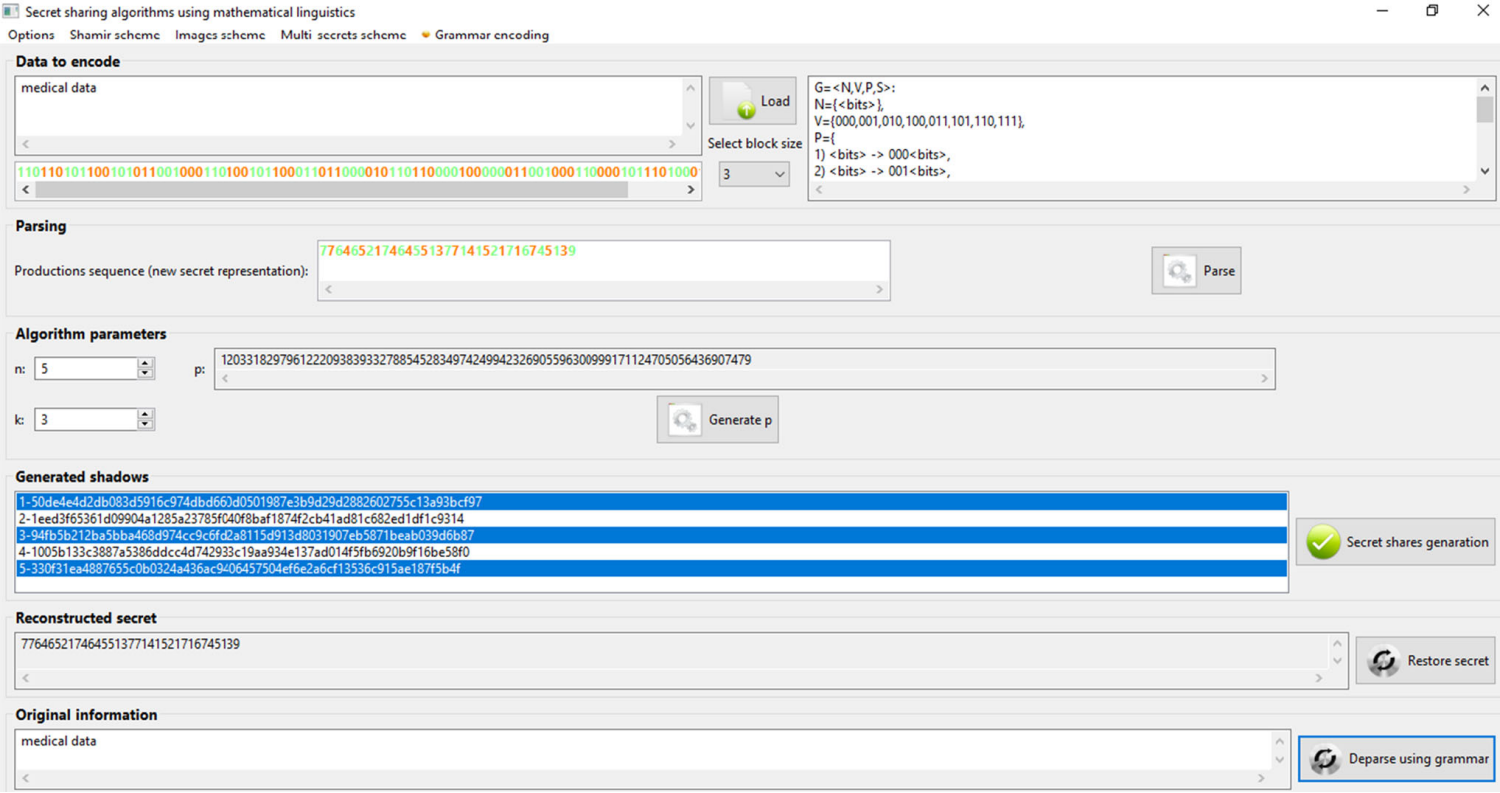
An example of linguistic threshold division using (3,5)—threshold schemes, and formal grammar

Application of linguistic formalisms in information division procedures extends the functionality of traditional information sharing schemes by generating an additional secret part having linguistic form (Fig. 2). Such additional parts will be necessary to reconstruct the original data.

Below is presented a generalized grammar formula for linguistic threshold procedure, which converts secret parts into independent bit blocks, which have the form of sequences of grammar production numbers. Such grammar representation can be defined in the following manner.

*{N*, *T*, *PS*, *STS}*, where *N* = (Data, Info_prart, Bit_sequence)—grammar non-terminal symbols; *T* = (bit, 2bit, 3bit, …, nbit, ε)—grammar terminal symbols; ε—an empty grammar symbol; *STS*—grammar start symbol; *PS*—grammar rules defined in the following manner:

1. Data $\to$ Info_prart Info_prart
2. Info_prart $\to$ Bit_sequence Bit_sequence | ε
3. Bit_sequence $\to$ bit | 2bit | 3bit …| nbit | ε

This grammar is a context-free sequential grammar for which efficient parsing algorithms exist, with polynomial computational complexity. Grammar defined in presented way enables the data to be divided in several different ways depending on the number of trusted users or requested security levels [10]. Example of data sharing using linguistic formula is presented in Fig. 2.

Presented linguistic solutions for data division extend classical security protocols and have important characteristic features. It allows to generate secret parts, available only for members of the authorized group. It also enhances traditional threshold procedures by adding a linguistic stage, at which binary representations of secret are coded into sequences representing the grammar rules. This causes that security of these techniques is independent of the length of encoded blocks, and complexity of the whole schemes remains polynomial.

## Supporting COVID classification using linguistic approach

Mathematical linguistics methods can also be used in the medical applications against the coronavirus pandemic states. Currently, many artificial intelligence methods are used in the detection and treatment of this viral disease [11, 12]. However, the appearance of different types of coronavirus makes it necessary to identify the type of infection that has a huge impact on the patient's prognosis and planning therapeutic actions.

The first coronaviruses were identified in the 1960s. There are four types of viruses, and they can be transmitted by humans, mammals and birds. Coronaviruses are viruses that contain only one strand of RNA nucleic acids in their structure. Despite the long-known structure of the coronavirus, 2019 saw a global epidemic caused by mutations of this virus. These mutations are still being discovered and differ from each other in their structural features, i.e., characteristic nucleic acid sequences in RNA strands composed of nearly 30,000 nucleotides. Their diagnosis is possible thanks to tests that recognize such structures.

Currently, several types of coronaviruses are defined that affect the course of the disease in a patient and the speed at which the infection spreads.

Three of them are currently being given special attention:

- British variant (name: VOC 202,012/01, lineage: B.1.1.7), which was detected in September 2020 in southern England. This variant is an accumulation of 17 previous mutations and is characterized by the fact that the infection lasts longer, i.e., about 13.3 days than in the case of the earlier variant, i.e., 8.2 days.
- South African variant (name: 501Y.V2, lineage: B.1.351,) that appeared in South Africa in January 2021 and is now detected in many other countries.
- Brazilian (name: P1, lineage: P1) variant that was identified in mid-2020.

Due to the differences in RNA strands, their classification is also possible using linguistic methods that allow to introduce the description of unique RNA structures using formal grammar. For this purpose, the following grammar can be defined to distinguish mentioned types of coronavirus based on the analysis of their RNA strands or its parts.

*{N*, *T*, *PS*, *STS}*, where *N* = (RNA, COV19-Type, British, African, Brazilian, N-Bases)—grammar non-terminal symbols; *T* = (A, C, G, U, ε)—grammar terminal symbols; ε—an empty grammar symbol; *STS*—grammar start symbol; *PS*—grammar rules defined in the following manner:

1. RNA $\to$ N-Bases COV19-Type N-Bases
2. COV19-Type $\to$ British | African | Brazilian
3. British, African, Brazilian $\to$ N-Bases N-Bases
4. N-Bases $$\to$$ N-Bases | A | C | G | U | ε

**Fig. 3:**
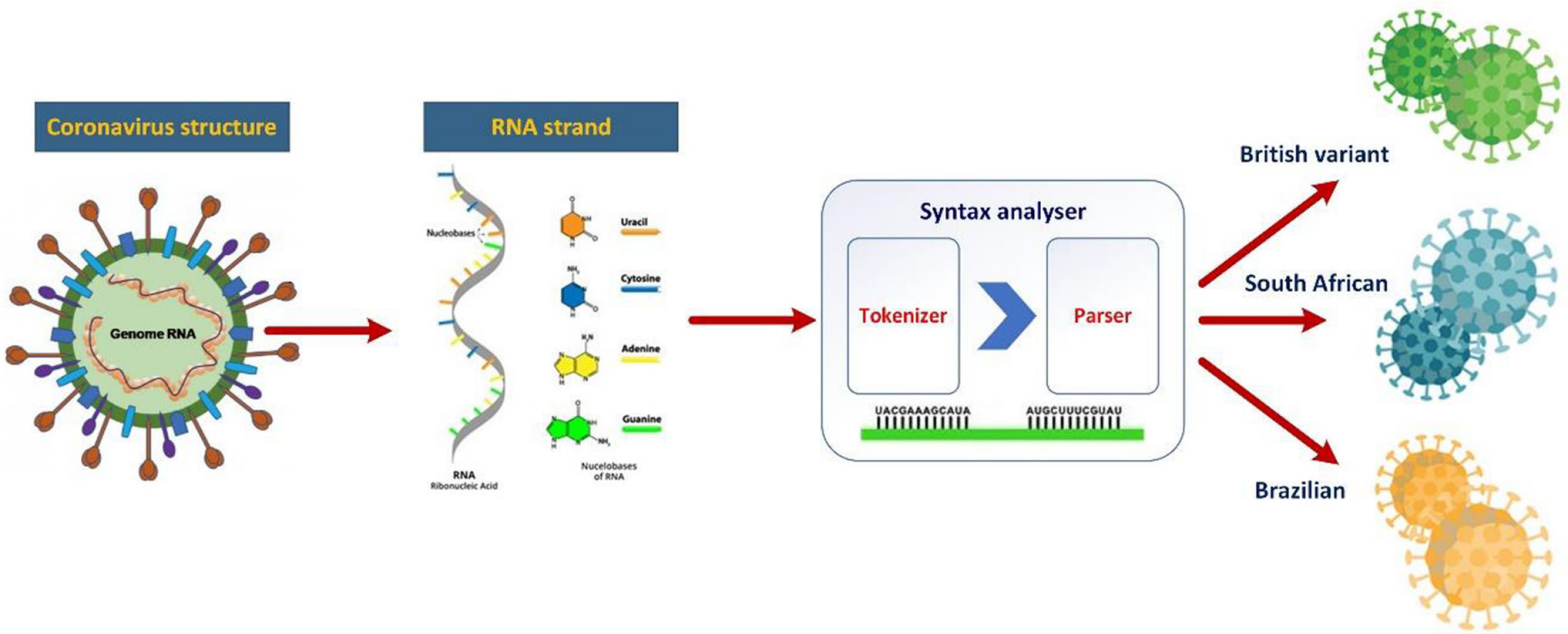
An example of syntax analysis of RNA strand linguistic representation using grammar parser

The proper analysis toward COVID-19 type identification can be done with application of syntax analyzer, which can parse the linguistic representation of the whole nucleic acid sequence or only its parts responsible for type of viruses. Example of such analysis is presented in Fig. 3.

During the analysis, the syntax analyzer tries to find special RNA sequences characteristic for particular coronaviruses variants. Special lexical analyzer called tokenizer allows to extract such unique RNA sequences and forward as input values for syntactic parser. Performing syntactic analysis parser can perform recognition of coronavirus variants, and place recognized case in one of predefined variants classes, i.e., British, South African, and Brazilian. In conducted experiments, it was possible to consider several cases representing different variants, which allows to define previously mentioned formal grammars and implement parser for syntactic analysis.

Because proposed grammar belongs to context-free class of formal grammars, we can implement for syntactic analysis parsers, which can perform top-down or bottom-up syntactic classification. The most common solutions in real application of syntax analyzers are implementation of reduction analysis (i.e., bottom-up) in which input pattern should be reduced by parser to the grammar start symbol.

Presented grammar can be easily extended toward description and recognition of others sequences defining future COVID-19 variants, by extension of grammar rules, and adding non-terminal symbols, which can be introduced especially for newly defined viruses.

## Conclusions

This paper describes the possibilities of using linguistic pattern recognition methods in medical applications. Linguistic methods are based on formal grammars and allow to describe complex medical structures and can also can be applied for security purposes and guarantee the security of medical data. Therefore, the paper describes two basic areas of using such techniques. The first one is application as intelligent methods for division of medical data, i.e., linguistic threshold schemes. The second important area of application is the analysis of disease states related to the detection of various mutations of the COVID-19 coronavirus. The paper defines a special context-free grammar that allows the classification of different variants of the coronaviruses, based on the analysis of selected fragments of the RNA strands. Such an analysis can support diagnostic processes related to the detection of disease states caused by the coronavirus, as well as support therapies thanks to information about the type of the detected disease variant [13].

Linguistic methods ca be also applied in many other areas connected with medicine and healthcare. One of the most important is classification of morphological changes of internal organs caused by disease processes. In such cases, linguistic methods allow to support diagnostic procedures by classification visible pathologies. In future, such techniques can be also implemented in intelligent cognitive systems, which imitate the natural processes of human thinking.
